# Boosting executive function in children aged 3–12 through musical training: a three-level meta-analysis

**DOI:** 10.3389/fpsyg.2025.1659927

**Published:** 2025-09-05

**Authors:** Yumeng Cai, Dan Kang, Xiwu Xu

**Affiliations:** Hunan Normal University, Changsha, China

**Keywords:** musical training, executive function, three-level meta-analysis, children, culture

## Abstract

For an extended period, musical training has been recognized as a key enhancer of children’s development, particularly affecting executive functions. This three-level meta-analysis examines the influence of musical training on executive functions in children aged 3–12 and identifies key moderating factors. The study analyzed 46 original research articles, encompassing 192 effect sizes from a total of 3,530 participants. The results demonstrate that musical training significantly enhances children’s executive function [*g* = 0.350, *p* < 0.001, 95% CI (0.247–0.453)]. The longer the duration and cycle, the greater the impact of musical training on children’s executive function. In collectivist countries, musical training is more effective. Among the sub-dimensions of executive function, inhibitory control showed the most significant improvement due to musical training. The discussion explores the theoretical and practical implications of these findings.

## Introduction

Executive functions (EFs) are a set of psychological operations aimed at guiding purposeful behavior toward specific goals (Diamond, 2013; Zelazo et al., 1997). These functions underpin the optimal cognitive, emotional, and social development of children (Shen et al., 2020) and are crucial during childhood, a key stage for their maturation (Diamond, 2016). Consequently, identifying factors that can enhance children’s EFs is a significant area of interest for researchers.

Musical training, in particular, is thought to have a unique influence on the development of children’s EFs (Moreno et al., 2011; Saarikivi et al., 2016; Slevc et al., 2016). This training, a complex sensorimotor activity, necessitates planning and monitoring processes (Palmer and Drake, 1997). It encompasses a variety of forms, including instrumental and vocal training, individual and group settings, and courses of different durations and frequencies (Schellenberg and Lima, 2024), all of which contribute to cognitive development in children. Musical training activates multiple sensory channels and mobilizes physical movements and fine motor skills, laying a solid foundation for cognitive development (Malambo et al., 2022; Shen et al., 2020; Slevc et al., 2016). Moreover, learning musical rules and remembering musical symbols during training can significantly enhance EFs (Frischen et al., 2019; Joret et al., 2017).

Some researchers argue that musical training fosters the growth of various aspects of EF in children, such as working memory and inhibitory control (Bolduc et al., 2021; Bowmer et al., 2018; Bugos and DeMarie, 2017; Chen et al., 2022; Moreno et al., 2011), with these effects being long-lasting (Shen et al., 2019). Neuroscience research has further shown that musical training can rapidly increase cortical thickness in the frontal lobes, closely associated with the growth of EFs (Hudziak et al., 2014), thus providing a physiological explanation for their correlation. However, other researchers contend that the impact of musical training on the sub-dimensions of children’s EFs is highly variable and influenced by numerous factors, including the content of the musical training intervention (Bowmer et al., 2018; Frischen et al., 2019; Janus et al., 2016).

This meta-analysis differs from previous studies on the effects of musical training on executive functioning in four key ways. First, it expands the scope of musical training to include instrumental, physical rhythmic, music mixing, and aural training, comparing these as moderating variables. This comprehensive approach offers a deeper understanding of how different types of musical training impact children’s EFs, an area that previous research, mainly focused on instrumental training (Román-Caballero et al., 2022), has not fully explored. Second, the study uses a three-level meta-analysis, accounting for interdependencies between multiple effect sizes. This method is statistically rigorous, reducing bias and errors, and provides a more accurate reflection of the overall impact of musical training on EF (Borenstein, 2013). Third, it includes children aged 3–12, offering insights into how EF develops across various age groups. In contrast, previous studies, like Lu et al. (2025), often focus on specific age ranges. Finally, the study examines all sub-dimensions of executive functioning—working memory, inhibitory control, and cognitive flexibility—providing a more nuanced and valid perspective. Previous meta-analyses have typically focused on a single dimension (e.g., inhibitory control in Jamey et al., 2024). Additionally, this analysis includes a wide range of sources, such as journals and theses, to minimize publication bias and statistical errors. In contrast, previous studies like Lu et al. (2025) only considered 10 studies, which increases the risk of bias.

### Musical training for children’s executive function

Why can musical training influence EFs? Several theoretical models offer explanations for this phenomenon Moreno and Bidelman (2014) introduced the Two-dimensional Transfer Model, which explains the impact of musical training on EFs from the perspective of cognitive transfer. They categorize transfer caused by musical training into near transfer (enhancement through music-related activities) and far transfer (enhancement through music-unrelated activities), as well as sensory transfer (enhancement of perceptual abilities) and cognitive transfer (enhancement at a more general cognitive level). They argue that musical training promotes EF growth from near to far and from sensory to cognitive levels.

Another explanatory model is derived from the generative theory of emotion (Ye et al., 2021). This theory posits that individuals actively and proactively perform cognitive assessments of their environment, a process termed “meaning construction.” In this dynamic process, emotions emerge as actions unfold and propel those actions forward. As children engage in musical training and interpret the musical environment, the music stimulates ongoing meaning construction, generating positive emotions and thus promoting EF growth.

From the perspective of internal physiological mechanisms, the “neuronal recycling” hypothesis suggests that neural networks adapt to new tasks by reusing effective networks and suppressing obsolete ones when acquiring cultural skills, processes known as “neuronal reuse” and “neuronal heuristics suppression” (Ahr et al., 2016; Dehaene and Dehaene, 2005). Neuroscience research has shown that the brain regions activated by musical training are closely related to EFs (Hudziak et al., 2014). Therefore, as children participate in musical training, the continuous emergence of new tasks in the training drives the iterative development of the neuronal networks associated with children’s EFs, thereby enhancing their development.

### Moderators

Previous research shows that different types of musical training affect EF sub-dimensions in children, with age playing a key role in the effectiveness of these interventions. According to Piaget’s developmental theory, the span from roughly 2–12 years encompasses the pre-operational and concrete-operational stages (Feldman, 2004). These stages coincide with the sensitive period for EF growth. Besides, early musical training can be woven naturally into children’s everyday routines and classroom activities, and is therefore likely to boost EFs more effectively during this window than in adolescence, when heavier academic demands may hinder such interventions. Empirical findings corroborate the advantage of early training (Chen et al., 2022; Diamond, 2013), potentially because music and EF neural networks interact synergistically (Bailey and Penhune, 2013) and because early practice accelerates dorsolateral prefrontal-cortex development (Hudziak et al., 2014). However, peak development times for different sub-dimensions vary: inhibitory control develops during preschool years (Shanmugan and Satterthwaite, 2016), working memory peaks around ages 7–9 (Lensing and Elsner, 2018), and cognitive flexibility develops during school years (Diamond, 2013). Thus, age may be a significant moderating factor in the effectiveness of musical training on EFs.

Music, as a cultural artifact, and the emotions it evokes can vary significantly across different cultures. Research has shown cultural differences in the pleasure derived from music; for example, the Tsimane people of the Amazon rainforest do not perceive dissonant tones as unpleasant, unlike other cultures (McDermott et al., 2016). The emotions evoked by music can significantly influence the mobilization and development of EFs, with negative emotions widely shown to impair the efficiency of EF operations (Zhou, 2013). Different emotional responses can also affect children’s cognitive evaluations and meaning construction of their environments (Ye et al., 2021), thereby impacting the development of EFs.

The content of training may affect the impact of musical training on children’s EFs. Embodied cognition theory suggests that cognitive processes, such as thinking, emotion, and motivation, are not merely symbolic processing within the brain but are products of the interaction between the brain, body, and environment (Ye, 2023). In children’s musical training, teachers employ a variety of teaching aids, activities, and environments to maintain children’s attention and facilitate their understanding of music. These diverse activities can impact children’s various mental processes, including EFs.

According to the memory theory proposed by Ebbinghaus, the frequency of repeated learning should be appropriate (Smolen et al., 2016). On one hand, too low a frequency may lead to excessively long intervals that fail to engage previous memories and experiences; on the other hand, too high a frequency may prevent these experiences from being reactivated and retrieved, thus inhibiting the enhancement of memory and cognition (Rubin, 1998; Tzeng et al., 1980). And the duration and cycle may also affect the intervention effect of musical training on children’s EF.

This research adopts the three-component model of EFs proposed by Diamond (2013), including inhibitory control, working memory and cognitive flexibility Existing research indicates that the impact of musical training on these components of EFs varies. Musical training is often considered more effective in promoting the development of children’s inhibitory control and working memory due to its inherent structural qualities (Diamond and Ling, 2016; Shen et al., 2019). Firstly, music has natural rules; children must restrain and adapt their behavior to comply with musical guidelines such as rhythm and melody and use their working memory to update and identify musical symbols (Joret et al., 2017; Okada and Slevc, 2018; Shen et al., 2019; Slevc et al., 2016). Secondly, musical training requires the integration of information from various senses, the suppression of unnecessary distractions, and the retention of this information in working memory (Moradzadeh et al., 2015). Thus, it might be concluded that musical training offers significant advantages in developing children’s inhibitory control and working memory.

### Current study

Given that research on musical training’s impact on children’s EFs includes multiple indicators (cognitive flexibility, working memory, and inhibitory control), the coding process often yields different effect magnitudes from an individual study. However, a core principle of conventional univariate meta-analyses is that effect magnitudes are unrelated, thus this study employs a triple-tiered model for addressing the dependencies among multiple effect magnitudes within individual studies (Cheung, 2021).

The purpose of this study was to quantitatively assess the existing literature on the effects of musical training on children’s executive functioning through a three-level meta-analysis. First, we aimed to investigate the effects of musical training on children’s executive functioning. We hypothesized that musical training would effectively promote the development of children’s EFs. Second, we investigated whether this association is affected by certain methodological, sample, and study characteristics, namely: children’s age, cultural background, the content of musical training interventions, frequency, duration, and weeks of intervention, and the specific sub-dimensions of EF. We hypothesized that the effects of musical training on children’s EFs would be influenced by these factors.

## Method

This research adhered to the Preferred Reporting Items for Systematic Reviews and Meta-Analysis Protocols (PRISMA-P) guidelines proposed by David et al. (2015).

### Search strategy

A comprehensive literature search from 1990 to 2025 was performed using Chinese databases such as China National Knowledge Infrastructure (CNKI) and English databases, including Web of Science, Springer, and Science Direct. The search involved identifying relevant papers using the following keywords in titles and abstracts: (1) “Musical Training” OR “Music” OR “Singing” OR “Instrument” OR “Dance” OR “Rhythm”; (2) “executive function” OR “inhibitory control” OR “inhibition” OR “cognitive flexibility” OR “flexibility” OR “working memory”; (3) “Preschooler” OR “Toddler” OR “Child.” The Chinese search terms were “音乐” OR “律动” OR “乐器” OR “舞蹈” AND “学前儿童” OR “幼儿” OR “儿童” AND “认知灵活性” OR “工作记忆” OR “抑制控制” OR “执行功能.” The reference lists of the identified papers were also reviewed to find additional sources.

### Inclusion criteria

**Figure 1:**
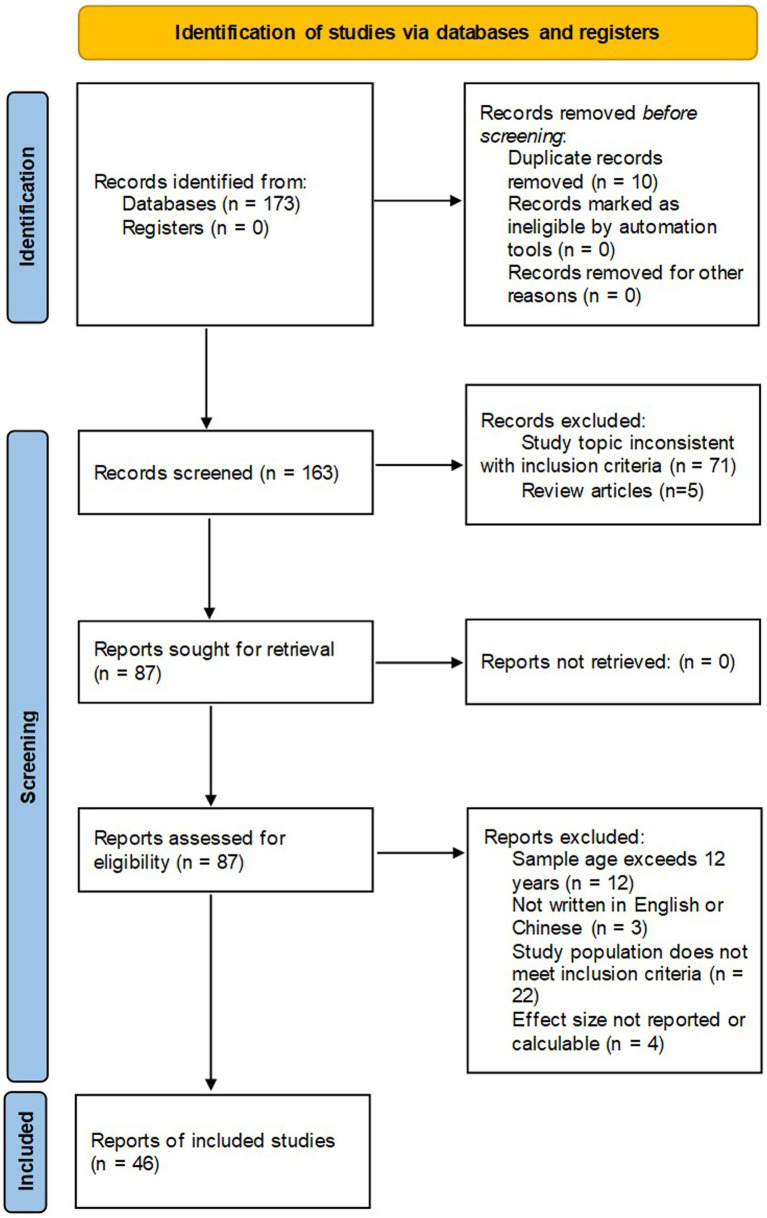
PRISMA diagram.

The inclusion criteria for the meta-analysis were as follows: (1) empirical studies, excluding meta-analyses, reviews, and qualitative research; (2) studies examining at least one core EF component (working memory, cognitive flexibility, inhibitory control); (3) studies involving typically developing children aged 3–12, excluding special populations (e.g., children with autism or ADHD); (4) interventions primarily involving musical training; (5) studies reporting quantifiable effect magnitudes (e.g., sample sizes, means, standard deviations, *t*-values, *F*-values) for both experimental and control groups; (6) studies with pretest and posttest measures; (7) studies published in Chinese or English. The PRISMA flow diagram is shown in Figure 1.

### Data extraction

Following the selection criteria described above, the articles included in the meta-analysis were coded with the following information:

1. basic bibliographic details (first author’s name, year of publication);
2. number of participants in the experimental and control groups;
3. age range of participants (3–6 years; 7–12 years);
4. weeks of intervention period, frequency of interventions per week, duration of one intervention;
5. sub-dimensions of EF studied (cognitive flexibility, inhibitory control, or working memory);
6. cultural context (individualistic vs. collectivistic). Drawing on previous research, countries with scores of 50 or above are classified as individualistic countries, while those with scores below 50 are classified as collectivist countries (Cheng et al., 2021);
7. content of musical training. Since most studies included multiple musical training elements in their music interventions, defining the primary element could lead to subjective coding. Therefore, we conducted a comprehensive coding of the musical training elements included in the intervention, including singing, movement, instrument, music theory, and aural training.

Data were extracted and organized using an Excel spreadsheet, following these principles: (1) each independent sample was coded as a separate effect size, and when multiple samples were reported, each was coded individually; (2) if data overlapped, the source with more comprehensive information was selected. The search and coding process was independently performed by two researchers, who reviewed each study, compared results, and resolved discrepancies through consultation, with a third researcher intervening when necessary.

To enhance interpretability, we created categorical bins for age, frequency (week/times), duration (min), and total program length (week). Because many studies reported only an age range (not a mean), we split age into 3–6 years and 7–12 years, including the preschool/kindergarten and primary-school stages common to most educational systems. The cut-points for frequency, duration, and program length were adapted from Lu et al., 2025’s meta-analysis on musical training, and their statistical suitability was confirmed with the Akaike Information Criterion (AIC) (Chang et al., 2019; Muggeo, 2020; Nakajima and Ninomiya, 2025; Burnham and Anderson, 2004). The final grouping scheme was:

Intervention frequency: low = 1–2 sessions/week; high = ≥3 sessions/week.
Session duration: short = 1–44 min; long = ≥45 min.
Program length: short = 1–10 weeks; long = > 10 weeks.

### Meta-analysis process

#### Effect size calculation

To assess the impact of musical training on children’s EFs, it was essential to compare the developmental outcomes in EF following musical interventions across various studies. This research employed CMA 3.0 and R version 4.3.3 software to conduct a three-level meta-analysis, selecting Hedges’ g—a corrected version of Cohen’s d—as the measure of effect size. Effect sizes were categorized as follows: 0.8 for a large effect, 0.5 for a moderate effect, and 0.2 for a small effect (Kallapiran et al., 2015). Most studies derived effect sizes using standard deviations, means, and sample sizes from control and experimental groups. A few studies converted correlation coefficients (*r*-values) and *F*-values to effect sizes using CMA 3.0. For tests of EF where lower scores indicated better outcomes, negative values were adjusted to positive values before analysis.

#### Model selection

Several studies included in this meta-analysis used multiple assessment tools to measure different components of EF, resulting in reports of multiple effect sizes within the same study. These reported effect sizes often originated from the same sample and were therefore correlated, challenging the traditional meta-analytical approach that assumes statistical data independence. The three-level meta-analysis overcomes this by partitioning the total variance into variance due to sampling error (Tier 1), variance among effect sizes extracted from the same study (Tier 2), and variance among effect sizes extracted from different studies (Tier 3) (Cheung, 2021). Compared to traditional meta-analytical methods, the three-level meta-analysis effectively manages the dependencies among effect sizes within the same studies, thus enhancing data integrity and statistical efficiency. Consequently, this study adopted a three-level random effects model to conduct primary effect tests, heterogeneity tests, moderation effect tests, and publication bias tests.

### Tests of heterogeneity and moderating effects

The three-level meta-analysis model facilitates the examination of three sources of variance: variance due to sampling error (Tier 1), variance among effect sizes extracted from the same study (Tier 2), and variance among effect sizes extracted from different studies (Tier 3) (Cheung, 2021). In this study, heterogeneity was assessed using the Q-test for overall heterogeneity, and one-tailed log-likelihood ratio tests were conducted to further identify the distribution of heterogeneity across Levels 2 and 3 (Gao et al., 2024). If heterogeneity was detected, it was categorized as low, moderate, or high based on I^2^ values of 25, 50, and 75%, respectively, following Higgins (2003). Additional tests to identify sources of heterogeneity involved examining moderating effects. Key moderating variables in this study included the age of the children, cultural background, content of training, frequency of musical training, and sub-dimensions of EF. To ensure the representativeness of the results from moderating effects, the study adhered to Card (2016) recommendation that each category of the moderating variables should include no fewer than five effect sizes.

### Control and testing for publication bias

Publication bias is the phenomenon where studies with significant results are more likely to be published (Rodgers and Pustejovsky, 2021). This selective dissemination can result in a published literature that does not comprehensively represent the entire body of research conducted in the field (Franco et al., 2014). To counteract the potential impact of this bias on the robustness of our findings, this study included both published journal articles and unpublished dissertations. We assessed the presence of publication bias using funnel plots and the Egger-MLMA regression method. Funnel plots serve as a preliminary visual check for publication bias, suggesting an absence of significant bias when the data points are symmetrically distributed and cluster toward the upper middle of the plot (Wei et al., 2017). Given that the effect magnitudes included in our analysis are not independent, the Egger-MLMA regression method offers a more reliable control for Type I errors than traditional methods (Rodgers and Pustejovsky, 2021). Due to the multiple correlated effect magnitudes reported in the studies of our current meta-analysis, we employed the Egger-MLMA regression method to evaluate publication bias. If publication bias is detected, the trim and fill method is applied to adjust for this bias (Duval and Tweedie, 2000).

### Sensitivity analysis

The effect magnitudes reported in the studies included in our meta-analysis on the impact of musical training on children’s EF range from −1.495 to 1.744, indicating substantial variability. This variability suggests that the meta-analysis results could be influenced by outliers, potentially leading to misleading statistical conclusions (Kepes and Thomas, 2018). To address this, we employed the Cook’s distance to assess the impact of outliers on our results and to ensure their robustness.

## Results

### Study characteristics

Through our literature retrieval, we included 46 articles in the meta-analysis. The total sample size across these studies was 3,530, with 192 effect magnitudes reported. The number of effect magnitudes per study varied from 1 to 20. The publication dates of the included articles ranged from 2011 to 2024 (see Table 1).

**Table 1:** Characteristics of included studies.

First author	Year	Region	Experimental group N	Control group N	Age (years)	Sub-dimension	Frequency(/week)	Duration (min)	Week	Training methods
Bayanova et al.	2022	Russia	47	47	3–6	WM/IC/CF	3	–	24	M; S
Bayanova et al.	2024	Russia	37	37	6–9	WM/IC	2	50	48	M
Bentley et al.	2023	Australia	112	101	3–6	WM/IC/CF	2	20	8	I; M
Bolduc et al.	2021	Canada	50	58	3–6	IC	–	40	19	M
Bowmer et al.	2018	United Kingdom	14	25	3–6	WM/IC/CF	1	40	8	I; M; A
Brown et al.	2022	United Kingdom	148	43	3–6	IC	2	30	12	I; M; S
Bugos et al.	2022	United States	34	29	3–6	WM	2	45	6	M; S
Bugos et al.	2017	United States	17	17	3–6	IC	2	45	10	I; M; A
Cai et al.	2023	China	32	32	3–6	WM/IC/CF	2	35	8	I; M; S; A
Degé et al.	2011	Germany	16	18	9–12	WM	2	–	2	I
Degé et al.	2022	Germany	11	14	3–6	IC	3	20	14	I; M; S
Ding	2015	China	24	25	9–12	IC	1	40	12	M
D’souza et al.	2018	Canada	24	25	6–9	WM/IC	5	120	3	I; M; S; A
Fasano et al.	2019	Italy	55	58	6–9	IC	1	135	10	I; T; M; S
Fernandes et al.	2022	Brazil	19	28	9–12	WM/IC	3	60	16	M
Frischen et al.	2019	Germany	27	23	3–6	WM/IC	3	20	20	I; M; S; A
Frischen et al.	2021	Germany	27	36	6–9	WM/IC	1	45	32	I
Guo et al.	2018	Japan	20	20	6–9	WM/IC	2	25	6	I; M; S
Habibi et al.	2018	United States	21	24	6–9	WM/IC/CF	–	–	–	I
Hallberg et al.	2017	United States	26	22	3–6	WM	6	30	–	I
Hennessy et al.	2019	United States	28	31	6–9	IC/CF	5	84	–	I; T; S
Holochwost et al.	2017	United States	135	130	9–12	WM/IC/CF	5	120	39	I
Ilari et al.	2021	United States	51	52	3–6	WM/CF	2	40	5	I; M; S
Janus et al.	2016	Canada	29	28	3–6	WM/IC	7	180	3	T; M
Jaschke et al.	2018	Netherlands	42	37	6–9	WM/IC	1	90	96	I; T
Joret et al.	2016	Belgium	30	31	9–12	IC	–	–	–	I
Kosokabe et al.	2021	Japan	48	32	3–6	WM/IC/CF	5	30	6	I; M; A
Lin et al.	2023	China	8	8	3–6	WM/IC/CF	1	45	16	I; M; S
Liu	2023	China	24	25	6–9	WM/IC/CF	2	35	12	M
Luan	2023	China	35	37	3–6	WM/IC/CF	2	30	6	I; T; M; S
Maroti et al.	2019	Hungary	13	13	6–9	WM/IC	4	45	34	M
Moreno et al.	2011	Canada	24	24	3–6	IC	10	60	4	T; A
Nie et al.	2022	China	34	30	6–9	WM	3	60	16	T; S
Qui et al.	2013	China	57	56	3–6	WM/IC/CF	5	30	40	I
Roden et al.	2014	German	25	25	6–9	WM	1	45	72	I; S
Rose et al.	2019	United Kingdom	19	19	6–9	WM/CF	–	–	–	I
Saarikivi et al.	2016	Finland	22/21	21/25	9–12	CF	–	–	–	I
Sachs et al.	2017	United States	14	17	6–9	IC	5	84	–	I
Shen et al.	2020	China	30	30	3–6	WM/IC/CF	3	45	12	M
Shen et al.	2019	China	30	31	3–6	WM/IC/CF	5	–	8	T; M
Sperling et al.	2023	United States	84	103	6–9	WM/CF	1	–	–	I
Suppalarkbunlue et al.	2023	Thailand	39	40	3–6	WM/IC/CF	3	45	8	I; M; S; A
Vazou et al.	2020	United States	22	17	6–9	IC	2	30	7	I; M
Williams et al.	2023	Australia	112	101	3–6	IC	2	20	8	I; M; A
Zhang	2021	China	25	23	9–12	IC	2	60	8	M
Zou	2021	China	20	20	3–6	WM/IC/CF	3	20	12	I; M; A

WM, Working memory; IC, Inhibitory control; CF, Cognitive flexibility; S, Singing; I, Instrument; M, Movement; T, Music Theory; A, Aural Training; The unit of training frequency is once a week.

**Figure 2:**
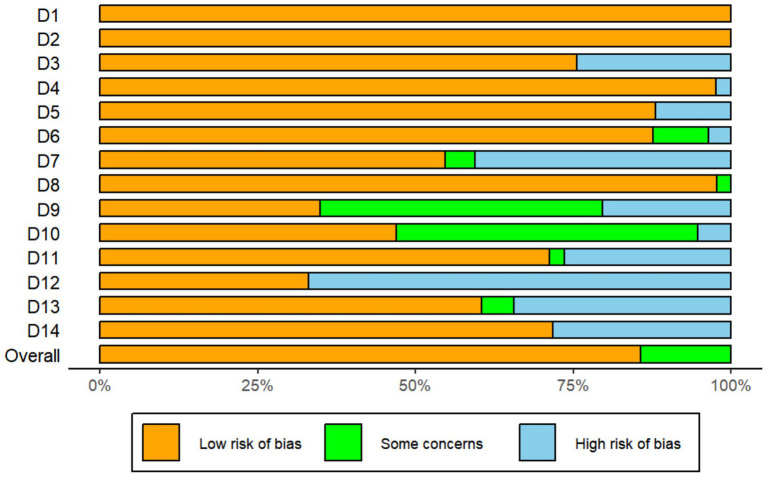
Risk of bias summary figure.

We systematically evaluated all 46 primary studies with the 2017 NIH Quality Assessment Tool for Observational Cohort and Cross-Sectional Studies (NIH, 2017). Two reviewers independently rated 14 methodological domains, resolving any disagreements by consensus. Overall, the studies showed high methodological quality and a low risk of bias; detailed ratings appear in the Risk of Bias Summary Figure (ROB) (see Figure 2).

### Main effect analysis and heterogeneity testing

This meta-analysis employed a three-level model to examine the main effects of musical training on children’s EF. The variance equation requires specifying a correlation value (rho) between the pre- and post-training values. As the published studies did not report this value, we imputed a value of rho = 0.5 when performing the meta-analyses. The results indicate that musical training positively influences the growth of children’s EFs, with an effect size [*g* = 0.350, *p* < 0.001, 95% CI (0.247–0.453)]. According to Kallapiran et al. (2015), this effect size is considered small. To make this effect easier to interpret, we compared it with other mainstream EF interventions. See the discussion section for details of these comparisons.

To assess overall variance heterogeneity, we utilized the Q-test. The *Q*-value from the triple-tiered meta-analysis model was 1489.341 (*p* < 0.001), indicating significant heterogeneity in the meta-analysis results. Further examination through the one-sided log-likelihood ratio test identified the distribution of this heterogeneity. The analysis revealed that the variability between effect magnitudes within the same study (Tier 2 variance) was 53.149%, and the variability between effect magnitudes across different studies (Tier 3 variance) was 30.622%, both of which were significant. According to Higgins (2003), these results suggest high intra-study heterogeneity and moderate inter-study heterogeneity. Consequently, it is essential to analyze moderating variables to further understand how musical training affects children’s EF. The results of the main effect analysis are displayed in Table 2.

**Table 2:** Main effect analysis of musical training on children’s executive function.

Model	N	*g*	95%*CI*		*Q*	*t*	Var. tier 1 *I^2^*(%)	Var. tier 2 *I^2^*(%)	Var. tier 3 *I^2^*(%)
Random effects	46	0.350	0.247	0.453	1489.341***	6.716***	16.230	53.149	30.622

N, number of studies;*p < 0.05, **p < 0.01, ***p < 0.001.

### Publication bias and sensitivity testing

**Figure 3:**
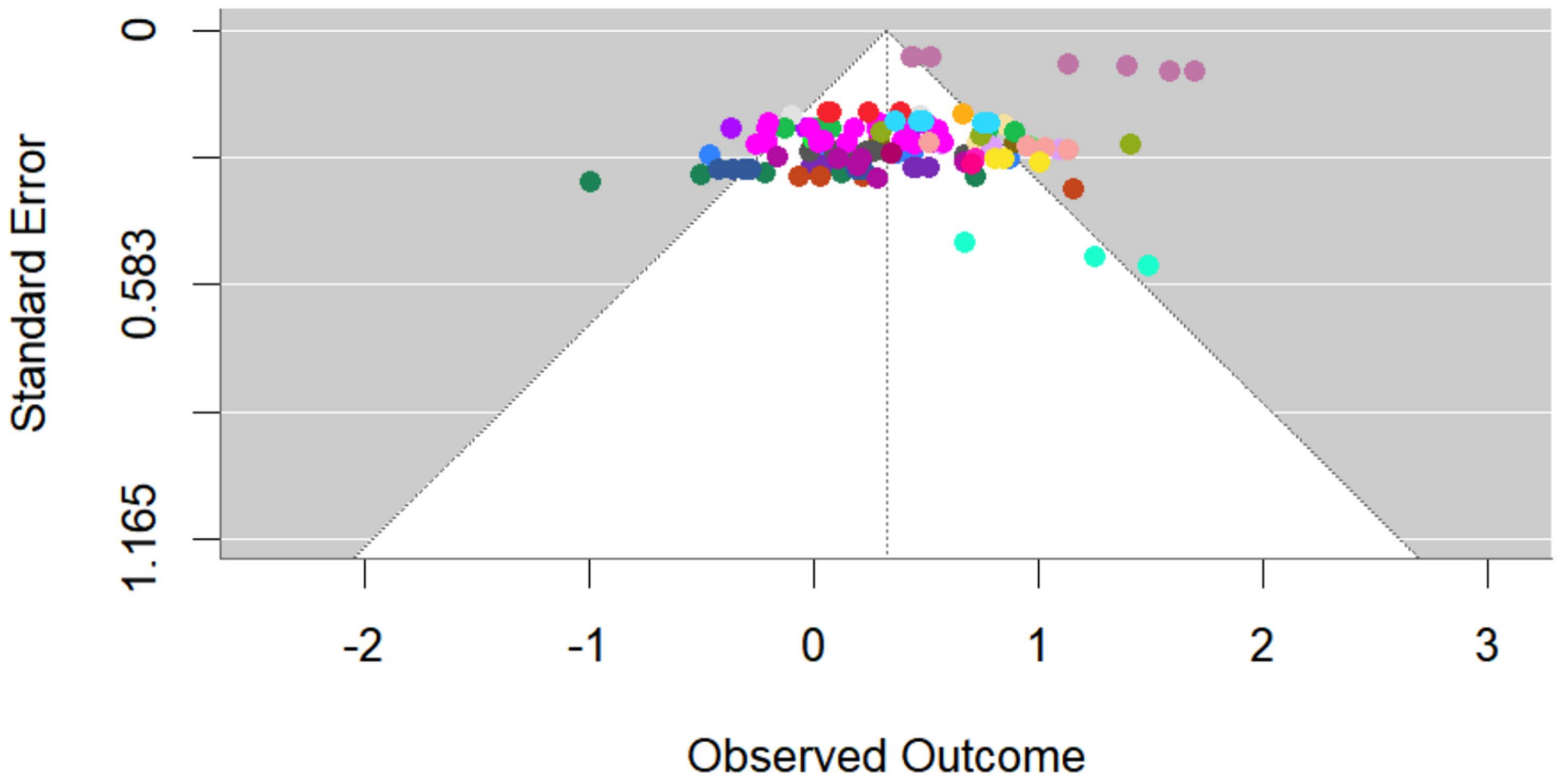
Funnel plot.

The Egger-MLMA regression results proved insignificant (*p* = 0.067), and the funnel plot exhibited symmetrical data distribution concentrated in the upper middle section. Dots of the same color represent data from the same study (see Figure 3). This indicates an absence of notable publication bias in the present meta-analysis. After conducting Cook’s distance analysis, six outliers were excluded from the effect size calculation to ensure the stability and reliability of the current meta-analysis results.

### Subgroup analysis by type of musical component

Moderated effects analyses require that each effect size can only be assigned to a single category. However, the vast majority of intervention programs in the included studies contained multiple music training components at the same time, which would inevitably result in category crossover if used directly as a moderating variable. To avoid this confusion, we first calculated the main effects separately for each component (see Table 3) and then compared the differences between the different components. The results showed that among the five components of “Singing, Rhythm, Aural Training, Music Theory, and Instrument,” Music Theory Training had the highest moderate effect size [*g* = 0.524, *p* < 0.001, 95% CI (0.271, 0.773)]; the rest were small effects, with Aural Training being the lowest [*g* = 0.231, *p* < 0.01, 95% CI (0.064, 0.397)]. The effect sizes for singing, rhythm and instrumental training were *g* = 0.398, *g* = 0.334, and *g* = 0.290, respectively (all *p* < 0.05; 95% CI are provided in Table 3).

**Table 3:** Subgroup analysis by type of musical component.

Intervention	N	g	95%*CI*		Q	t	Var. tier 1	Var. tier 2	Var. tier 3
							I2(%)	I2(%)	I2(%)
Singing	18	0.398	0.291	0.505	168.203***	7.432***	52.046	32.536	15.418
Movement	30	0.334	0.213	0.454	607.255***	5.462***	21.217	57.443	21.340
Aural training	8	0.231	0.064	0.397	87.083***	2.791**	50.914	31.041	18.046
Music theory	8	0.524	0.271	0.773	136.032***	4.236***	16.864	40.755	42.381
Instrument	32	0.290	0.166	0.413	1329.612***	4.637***	15.118	57.685	27.197

N, number of studies;*p < 0.05, **p < 0.01, ***p < 0.001.

### Examination of moderating effects

The results of the moderation effect tests are presented in Table 4. The impact of age [*F*(1,190) = 0.147, *p* = 0.702] and frequency [*F*(1,179) = 2.459, *p* = 0.119] were found to be insignificant. However, the effects of duration [*F* (1,167) = 5.472, *p* = 0.021] and training weeks [*F* (1,180) = 3.988, *p* = 0.047] were significant, suggesting that musical training with longer duration and extended cycles significantly enhances children’s EF. Furthermore, the effect of culture was significant [*F* (1,190) = 4.498, *p* = 0.035], indicating that musical training is more effective in collectivist countries. The analysis of the sub-dimensions of EF also yielded significant results [*F* (2,189) = 5.481, *p* = 0.005], with musical training having the most substantial effect on inhibitory control [*g* = 0.467, 95% CI (0.338, 0.596)], followed by working memory [*g* = 0.298, 95% CI (0.166, 0.430)], and the smallest impact on cognitive flexibility [*g* = 0.198, 95% CI (0.033, 0.364)].

**Table 4:** Tests of moderating effects of musical training on children’s executive function.

Moderator variable	K	#ES	Hedges’g	95%*CI*	Omnibus test	*p*	Var. tier 2 Var. tier 3
Age					*F*(1, 190) = 0.147	0.702	0.110***
							0.065***
3–6	24	116	0.332***	0.192, 0.473			
6–12	23	76	0.373***	0.219, 0.527			
Cultures					*F*(1, 190) = 4.498	**0.035**	0.109***
							0.058***
Individualism	32	112	0.270***	0.146, 0.395			
Collectivism	15	70	0.496***	0.327, 0.666			
Frequency (week/times)					*F*(1, 179) = 2.459	0.119	0.115***
							0.064***
1–2	22	68	0.273***	0.117, 0.429			
≥3	20	113	0.447***	0.294, 0.599			
Duration (min)					*F*(1, 167) = 5.472	**0.021**	0.125***
							0.053**
1–44	19	89	0.235**	0.078, 0.392			
≥45	21	80	0.491***	0.342, 0.640			
Weeks					*F*(1, 180) = 3.988	**0.047**	0.116***
							0.055***
1–10	20	85	0.262***	0.112, 0.413			
>10	24	97	0.471***	0.330, 0.613			
Sub-dimension					*F*(2, 189) = 5.481	**0.005**	0.097***
							0.075***
Inhibitory control	36	79	0.467***	0.338, 0.596			
Working memory	32	75	0.298***	0.166, 0.430			
Cognitive flexibility	21	38	0.198*	0.033, 0.364			

For each moderator, the reference category is listed first. #ES, number of effect magnitudes; K, number of studies; *p < 0.05, **p < 0.01, ***p < 0.001. Bold values represents *p* < 0.05.

## Discussion

This study conducted a triple-tiered meta-analysis, synthesizing data from 46 studies with 3,530 participants and 192 effect sizes. The results show that musical training significantly enhances children’s EFs [*g* = 0.350, *p* < 0.001, 95% CI (0.247–0.453)]. The effect size we observed exceeds that of non-computerized games (*g* = 0.30) and physical-activity programs (*g* = 0.16) designed to enhance children’s EFs, and is only marginally below the gains reported for mindfulness meditation (*g* = 0.46) and computerized EF training (*g* = 0.42) (Takacs and Kassai, 2019). Taken together, these comparisons indicate that musical training offers an appealing compromise between practical suitability for children and demonstrable cognitive efficacy. Music is distinguished by its pronounced temporal structure, which calls for rhythm-based prediction (Friston, 2010); its abstract symbolic language of notation; and its exacting demand for real-time auditory–motor coordination (Patel, 2011a; Tierney and Kraus, 2013). Collectively, these features may grant musical training a unique edge in enhancing EFs (Miendlarzewska and Trost, 2014), though rigorous comparative studies are still needed to confirm this advantage.

Duration, training weeks, culture, and specific EF components were found to moderate this effect, while age, training content and frequency did not. These findings support the two-dimensional transfer model, the generative theory of emotion, and the neuronal recycling hypothesis. Musical training positively influences children’s EF, thereby enhancing the explanatory power of the two-dimensional transfer model regarding the mechanisms involved (Moreno and Bidelman, 2014). Musical training engages children’s senses in multiple ways, enhancing their perceptual abilities. Children recognize pitch and rhythm through hearing, read music scores with their eyes, sing with their mouths, play instruments with their hands, and move rhythmically. This multisensory experience forms the foundation for developing cognitive aspects of EF.

These results align with emotion-generation theory, highlighting the way musical training nurtures children’s EFs by engaging their emotional systems. Melodies uniquely stimulate and modulate a network of emotion-related brain regions. Pleasant, soothing music, for instance, robustly activates the hippocampus—a key node for social bonding and stress regulation (Koelsch, 2014). As children immerse themselves in music, dopamine rises, stress diminishes, attention sharpens, and intrinsic motivation grows, together fostering cognitive development (Shen et al., 2019).

This study also supports the “neuronal recycling” hypothesis (Ahr et al., 2016; Dehaene and Dehaene, 2005), revealing the physiological basis by which musical training promotes children’s EF growth. In musical training, the neural networks and brain regions highly related to EF are developed (Hudziak et al., 2014), resulting in greater convergence between the neural networks used for musical training and those used for EF tasks. This leads to a reduction in obsolete “neuronal heuristics” and an increase in the “neuronal reuse” process. For instance, the rhythmic structure of music creates an ideal arena for honing inhibitory control. To stay in time—whether while playing an instrument or moving to a beat—children must precisely anticipate each pulse and suppress impulsive reactions (Friston, 2010; Patel, 2011b; Tierney and Kraus, 2013). Repeated practice recruits and strengthens the fronto-basal ganglia circuits that underlie inhibition control, helping to explain why musical training so reliably boosts this EF.

### Moderating variables

The moderating effect of children’s age was insignificant; thus, the Hypothesis was not supported. Although some studies suggest that the early childhood years are crucial for nurturing the growth of personal EF (Diamond and Ling, 2016), other studies have shown that enhancements in the auditory cortex and neurophysiological functions among musicians are positively associated with the length of ongoing training and inversely associated with the age when musical training begins (Zendel and Alain, 2013). This implies that the longer a person engages in musical practice and the earlier they start, the greater the advantages of musical training on the brain’s cortex and cognitive system. For preschool-aged children, the sensitive period for cognitive development provides favorable conditions for EF growth; however, for children of school-going age, their existing musical experiences also increase the likelihood that musical training will beneficially impact EF. The results of this study also demonstrate that the overall migratory effect of musical training on executive function is more stable at the temporal level under the two-dimensional transfer model.

Children’s musical training often involves various methods to engage their interest and promote overall development. Our grouping results indicated that among the five categories of singing, movement, aural training, and music theory and instrument, music theory training had the most significant enhancement of children’s executive functioning, with a medium effect size (*g* = 0.524) that was significantly higher than the small effects of the remaining four categories. Aural training had the weakest effect (*g* = 0.231). The reason why music theory training is so important is, on the one hand, because of its highly symbolic knowledge system—when learning notes, rhythms, and harmony rules, children need to continuously use inhibitory control and working memory to maintain and manipulate these abstract representations (Cara, 2024). On the other hand, compared to singing or playing an instrument, which require simultaneous processing of sound and movement, learning music theory has a lower cognitive load, allowing more cognitive resources to be focused on attention mobilization and rule prediction (Endestad et al., 2020). However, based on embodied cognition theory (Ye, 2023), auditory training, due to insufficient active physical movement and reliance on passive listening, has limited transfer effects on executive function. Given the significant differences in the contributions of various components to executive function, future music curriculum design should incorporate more elements that reflect embodied cognition while ensuring fun, and reasonably control cognitive load and emotional experiences to provide children with a comprehensive training environment that combines motivational value and cognitive challenges.

Musical training in collectivist countries has a stronger effect on children’s executive functioning than in individualistic countries, supporting the Hypothesis. This phenomenon can be attributed to the cultural emphasis on group goals, collaboration, and social relationships in collectivist societies (Chailitilerd, 2014). In these cultures, children are more engaged in cooperative activities such as ensemble performances or group dances. For example, highly structured mixed music training conducted in small groups in China, or MMT courses conducted in classrooms in Thailand to monitor children’s progress in music lessons, have all had an positive impact on children’s executive functioning (Shen et al., 2019; Suppalarkbunlue et al., 2023). These activities require constant self-regulation, inhibitory control, and memorization of new tunes or movements to align with the group, all of which are closely tied to EFs. As a result, musical training is more effective at enhancing EFs in collectivist countries, aligning with previous research that suggests collective cultural environments, particularly in East Asian cultures, foster the development of EFs (Leslie et al., 2017).

The effects of musical training on children’s EFs are mainly influenced by the duration of each training session and the total training period, while weekly training frequency has a more limited impact. Specifically, sessions of≥45 min delivered over > 10 weeks produced the strongest EF gains. For example, 34 weeks of 45-min music-based movement training or 16 weeks of 60-min music-based mixed training both achieved good effect sizes (Maróti et al., 2019; Nie et al., 2022). Longer training duration can engage children in deep cognitive processing, activating brain regions like working memory and attentional control, which enhances EF. Additionally, long-term musical training is more likely to improve brain structure through neural remodeling, thus benefiting EF, while short-term training may have less impact (Bialystok et al., 2012; Hyde et al., 2009). While the spacing effect can enhance learning efficiency, excessive training frequency may lead to attentional fatigue, reducing its benefits (Risko et al., 2016). Overall, the total training duration is more strongly linked to improvements in EF than frequency. The pragmatic dosage benchmark distilled here—45-min sessions sustained for at least 10 weeks—offers clear guidance for future educational practice.

The moderating effects of the sub-dimensions of EF were significant, supporting the Hypothesis. First, musical training requires children to control and adjust their behavior according to musical rules and to utilize working memory to understand musical notation (Malambo et al., 2022; Shen et al., 2019). Conversely, enhancing cognitive flexibility requires children to demonstrate more creativity during training, which is less emphasized in traditional musical training. Secondly, since the sensitive period for the development of inhibitory control occurs earliest (Shanmugan and Satterthwaite, 2016), and working memory develops more slowly (Davidson et al., 2006), while cognitive flexibility largely depends on the other two components and often develops during adolescence and adulthood (Moradzadeh et al., 2015; Saarikivi et al., 2016), in our study sample of children aged 3–12, enhancements in inhibitory control and working memory due to musical training were more frequently reported. In summary, the moderating effects of the sub-dimensions of EF were significant, and musical training had a stronger impact on inhibitory control and working memory, aligning with our initial hypotheses.

### Limitations and Future Directions

This research has several limitations: First, the meta-analysis only included studies involving children with typical developmental profiles, overlooking the specific effects of musical training on EF development in children with developmental challenges, such as autism or ADHD. This exclusion may have prevented a comprehensive inclusion of all potential child study samples in the meta-analysis. Future research should explore how musical training impacts EFs and other cognitive aspects in children with developmental disorders. Second, the wide variety of EF assessment tools—and the sparse reporting of intervention “dose” variables—constrained the depth of our moderator analyses. Most primary studies described “frequency” merely as sessions per week, rarely clarifying spacing patterns (e.g., daily brief sessions vs. weekly massed sessions) or total instructional minutes. We therefore urge future researchers to (a) report a full suite of dose descriptors—sessions per week, session length, total minutes, and program duration—and (b) adopt or develop harmonized, age-appropriate EF batteries to enable cross-trial comparison. Drawing on our data, we also derived and empirically validated several pragmatic dichotomous cut-points; subsequent work should test the stability of these thresholds through finer-grained subgrouping or continuous-variable modeling. Lastly, because the available evidence base is largely cross-sectional or short-term, additional longitudinal research is needed to track the durability of training-related EFs gains and to clarify how various moderating variables operate over time. We therefore advocate prospective cohort or repeated-measures designs that follow children months or years post-intervention, using standardized EF test batteries to facilitate meta-analytic synthesis and cumulative knowledge building.

## Conclusion

This research, utilizing a three-level meta-analysis approach, found that musical training enhances children’s EF and provided a theoretical explanation of the results from three perspectives: cognitive transfer, emotional dynamics, and internal physiological mechanisms. The relationship between musical training and EF is influenced by the culture, duration and week of training and the components of EF. Although the overall effect size is modest, it compares favorably with many other intervention programs and is paired with high acceptability among children and excellent scalability. Consequently, musical training represents a promising avenue for enhancing EF development.
