# Correction: Boosting executive function in children aged 3–12 through musical training: a three-level meta-analysis

**DOI:** 10.3389/fpsyg.2026.1773874

**Published:** 2026-01-20

**Authors:** Yumeng Cai, Dan Kang, Xiwu Xu

**Affiliations:** Hunan Normal University, Changsha, China

**Keywords:** musical training, executive function, three-level meta-analysis, children, culture

Funding was erroneously omitted from the published article.

The correct Funding appears below:

The author(s) declared that financial support was received for this work and/or its publication. This research was supported by the Education Department of Hunan Province (Hunan Provincial Department of Education) [23A0066] to Dan Kang.

The original version of this article has been updated.

